# Intranasal Delivery of Influenza rNP Adjuvanted with c-di-AMP Induces Strong Humoral and Cellular Immune Responses and Provides Protection against Virus Challenge

**DOI:** 10.1371/journal.pone.0104824

**Published:** 2014-08-20

**Authors:** Maria Victoria Sanchez, Thomas Ebensen, Kai Schulze, Diego Cargnelutti, Paulina Blazejewska, Eduardo A. Scodeller, Carlos A. Guzmán

**Affiliations:** 1 Laboratory of Virology, Institute of Experimental Medicine and Biology of Cuyo (IMBECU-CCT, CONICET), Mendoza, Argentina; 2 Department of Vaccinology and Applied Microbiology, Helmholtz Centre for Infection Research, Braunschweig, Germany; 3 Boehringer Ingelheim Veterinary Research Center GmbH & Co. KG, Hannover, Germany; National Center for Cell Science, India

## Abstract

There is a critical need for new influenza vaccines able to protect against constantly emerging divergent virus strains. This will be sustained by the induction of vigorous cellular responses and humoral immunity capable of acting at the portal of entry of this pathogen. In this study we evaluate the protective efficacy of intranasal vaccination with recombinant influenza nucleoprotein (rNP) co-administrated with bis-(3′,5′)-cyclic dimeric adenosine monophosphate (c-di-AMP) as adjuvant. Immunization of BALB/c mice with two doses of the formulation stimulates high titers of NP-specific IgG in serum and secretory IgA at mucosal sites. This formulation also promotes a strong Th1 response characterized by high secretion of INF-γ and IL-2. The immune response elicited promotes efficient protection against virus challenge. These results suggest that c-di-AMP is a potent mucosal adjuvant which may significantly contribute towards the development of innovative mucosal vaccines against influenza.

## Introduction

Seasonal vaccination against influenza is the best available measure to reduce the high impact of this pathogen on public health worldwide. However, the immunity conferred by current vaccines is sub-optimal. For example: i) the degree of coverage is low and variable and seasonal vaccine only protects against viruses which share a high degree of homology with the original vaccine strain [1], [2] ii) most vaccines are administered parentally, leading to unsatisfactory mucosal responses [3], [4], and iii) the elicitation of cell mediate immunity is very poor or absent [5], [6].

The efficacy of current vaccines is mainly based on the induction of neutralizing antibodies that target the surface viral protein hemagglutinin (HA). This protein is subjected to a high degree of antigenic variation and new divergent strains continuously arise in nature, which are not recognized by antibodies induced by the vaccine. As a consequence, the vaccine seed stocks need to be updated every year [7]. To overcome this limitation, efforts were invested in the development of a vaccine that can protect against the multiple antigenic variants, a concept that is known as “universal vaccine” [8]. This concept is based on the use of conserved antigens, such as the amino terminus of the M2 protein (M2e or M2 ectodomain) [9], the conserved stalk domain of the HA [10], the M1 protein and the nucleoprotein (NP) [11], [12].

The strategy based on the use of M1 or NP as antigens is based on the elicitation of strong cell-mediated immunity rather than the induction of neutralizing antibodies. Such approach has been termed as "T-cell vaccine" [13], [14]. In this regard, it has been recognized for over 30 years that adoptive transfer of lymphocytes from influenza infected mice to naïve animal confer protection to recipients against different viral strains, the so-called heterotypic immunity [15], [16]. It was also demonstrated that the NP represents a major recognition target [17]–[19]. Although there are several other viral proteins bearing T-cell epitopes able to induce this type of immunity, NP stands as one of the most conserved and effective target antigen [20], [21]. Numerous studies showed that immunization with recombinant NP can confer protection against experimental infection with divergent viruses. In some cases protection was principally mediated by CD4+ and by CD8+ T cells [22]–[28]. Unexpectedly, recent studies have shown that non-neutralizing anti-NP antibodies elicited in vaccinated animals can also confer protection against influenza infection [29]–[32].

Up to now, the most effective strategy to obtain protection in vaccinated animals using conserved antigens has been the generation of recombinant viral vectors (e.g. adenoviruses or poxviruses) expressing M1 and NP proteins [11], [12], [33]. The vaccinia virus based approach has been successfully tested in human trials [12], [13]. However, the use of live vectors (*e.g*. adenoviral vectors) for mass vaccination campaigns is associated with potential safety issues [34].

Furthermore, another limitation of most current available vaccines is that they are administered by the intramuscular route, thereby not leading to the stimulation of mucosal immune responses. There is only one intranasal influenza vaccine in the market which is based on a live attenuated virus (FluMist, MedImmune, USA). However, mucosal vaccination could establish a first line of defense at the mucosal tissues, the portal of entry, for this important pathogen [35], [36]. Thus, important features of a new generation influenza vaccine would be their capacity to induce strong humoral and cellular immune responses even at mucosal territories [35]–[37]. The combination of a conserved influenza antigen with an efficient mucosal adjuvant could be a good approach to achieve this goal [35], [36], [38]–[40].

We observed in previous studies that the bis-(3′,5′)-cyclic dimeric adenosine monophosphate (c-di-AMP) combined with model antigens, such as OVA or β-Gal, acts as a potent mucosal adjuvant stimulating both humoral and cellular responses [41]. Here, in the present work we demonstrated that intranasal administration of a rNP influenza vaccine with c-di-AMP, results in the stimulation of both humoral and cellular specific immune responses at both systemic and mucosal levels, which confer efficient protection against influenza viral challenge.

## Material and Methods

### Immunization schedule and vaccines

Groups of female BALB/c (H-2d) mice (n = 5) of 6 to 8 weeks of age purchased from Harlan Winckelmann GmbH (Borchen, Germany) were immunized on days 0 and 21 by intranasal route. The recombinant influenza nucleoprotein (rNP), derived from the influenza strain A/PR/8/34 (H1N1), was synthesized and purified as previously described [42]. The synthesis and purification of the mucosal adjuvant c-di-AMP was described in Ebensen et al [41]. For vaccine preparation, lyophilized c-di-AMP was freshly dissolved in distilled water. Mice received 10 µg/dose of rNP alone or co-administered with 10 µg/dose of c-di-AMP as adjuvant. A negative control group received phosphate buffered saline (PBS). The solutions were prepared in PBS 30 min before administration (final volume of 20 µl; 10 µl per nostril). To facilitate animal manipulations mice were shortly anesthetized with Isofluorane (Abbott Animal Health), according to the manufacturer's instructions. Animals were breed at the animal facility of the Helmholtz Centre for Infection Research under specific pathogen free conditions with food and water *ad libitum*. All animal experiments in this study have been performed in agreement with the local government of Lower Saxony (Germany), under the animal permission code No. 33.11.42502-04-017/08.

### Sample collection

Blood samples were collected on days 0, 21 and 42 via retro-bulbar bleeding. The samples were incubated for 60 min at 37°C and for 30 min at 4°C, then centrifuged to remove red blood cells (5 min at 8,000×g) and sera were stored at −20°C until processing. Then, mice were sacrificed and broncho-alveolar (BAL) and nasal lavages (NL) were obtained by flushing the local tissues with ice-cold PBS supplemented with 5% FCS (Greiner Bio-One, USA) and 40 µM phenyl-methane-sulfonyl-fluoride (PMSF). Lavages were centrifuged (5 min at 8.000 g) to remove debris and stored at −20°C until processing for the detection of immunoglobulin A (IgA). Cells from spleen were collected and monitored for the presence of antigen-specific T and B lymphocytes. Subsequently spleen was taken aseptically and disaggregated in complete RPMI (RPMI 1640 supplemented with 10% fetal calf serum, 100 U/ml penicillin, 50 µg/ml streptomycin, 100 µg/ml gentamycin; Gibco, USA). For the depletion of the erythrocytes the pellet was re-suspended in ACK lysis buffer. After washing, cell suspensions were cultured in complete RPMI and used for the determination of cellular immune responses. Cellular responses were analyzed using pools of spleen cells, whereas antibodies were examined by investigating individual animals.

### Elispot assays

To determine gamma interferon (IFN-γ), interleukin-2 (IL-2), interleukin-4 (IL-4) and interleukin-17 (IL-17) secreting cells, murine IFN-γ, IL-2, IL-4 and IL-17, enzyme-linked immunospot (ELISPOT) kits (BD Pharmingen, USA) were performed, according to the manufacturer's instructions. Cells (1×10^6^ or 5×10^5^ cells/well) were incubated for 24 h (IFN-γ) or 48 h (IL-2, IL-17 and IL-4) at 37°C with 5% CO_2_, in the absence or presence of 2 µg/ml of rNP, or an immune-dominant MHC class I restricted peptide (NP 147–155) or a mix of MHC class II restricted peptides (NP 182-205, NP 55-77 and NP 206-229), which were synthesized at the HZI. After 24 or 48 h, cells were removed and the plates were processed according to the manufacturer's instructions. Colored spots were counted with an ELISPOT reader (CTL S5 Micro Analyzer) and analyzed using ImmunoSpot image analyzer software v3.2 (CTL Europe GmbH, Germany).

### Determination of NP-specific IgG in serum

The NP-specific antibodies were determined by enzyme-linked immunosorbent assay (ELISA) using microtiter plates coated with 100 µl/well of rNP (2 µg/ml in carbonate buffer; pH 8.2). After overnight incubation at 4°C, plates were blocked with 3% BSA in PBS for 1 h at 37°C. Serial two-fold dilutions of sera in 1% BSA–PBS were added (100 µl/well), and plates were incubated for 2 h at 37°C. After six washes with 3% BSA–PBS–0.05% Tween 20, secondary antibodies were added: biotinylated chain-specific goat anti-mouse IgG (Sigma, USA) or, to determine IgG subclasses, biotinylated rat anti-mouse IgG1, IgG2a and IgG3 (BD Pharmingen, USA). Plates were further incubated for 2 h at 37°C. After six washes, 100 µl/well of peroxidase conjugated streptavidin (BD Pharmingen, USA) was added to each well, and plates were incubated at room temperature for 1 h. After another six washes, reactions were developed using ABTS [2,2-azinobis(3-ethylbenzthiazoline-6-sulfonic acid)] in 0.1 M citrate-phosphate buffer (pH 4.35) containing 0.01% H_2_O_2_. End-point ELISA titers were expressed as the reciprocal of the highest sample dilution that yielded an OD ≥2 times above the mean value of the blank.

### Determination of NP-specific IgA in mucosal lavages

The amount of antigen-specific IgA presents in the NL and BAL were determined by ELISA, as previously described by Ebensen et al. [41]. Briefly, 96 well microtitre plates were coated with 100 µl per well of rNP in a concentration of 2 µg/ml, diluted in carbonate buffer and incubated for 2 h. After blocking with 3% BSA/PBS for 1 h at 37°C, the plates were washed and further incubated with 2-fold serial diluted lavages samples for 1 h at 37°C. After 6 washes, biotinylated alpha-chain specific goat anti-mouse IgA (Southern Biotech, USA) was added and plates were further incubated for 1 h at 37°C. After 6× washes, the peroxidase-conjugated streptavidine (BD Pharmingen, USA) was added and the plates were incubated at RT for 1 h. The plates were washed and reactions were developed using ABTS in 0.1 M citrate–phosphate buffer (pH 4.35) containing 0.01% H_2_O_2_. To compensate variations in the efficiency of recovery of secretory antibodies among animals, the results were normalized and expressed as endpoint titers of antigen-specific IgA per µg of total IgA present in the sample. In brief, plates coated with 2 µg/ml of goat anti-mouse IgA (Sigma) as capture antibody were incubated with serial twofold dilutions of either lavage samples or, for the standard curve, purified mouse IgA (Dianova, Hamburg, Germany) for 1 h. After serial washes with PBS plus 0.1% Tween 20, plates were incubated for 1 h with the secondary antibody, biotinylated goat anti-mouse IgA (Southern Biotech, USA), washed six times, and developed as described above.

### Proliferation

Pooled spleen cells (5×10^5^ cells/well) of each vaccination group were incubated in quadruplicates for 96 h in the presence of different concentrations of, rNP (0.1, 1 and 2 µg/ml), a peptide encompassing a MHC class I-restricted epitope (NP 147-155) or a pool of peptides spanning different MHC class II-restricted epitopes (NP 182-205, NP 55-77 and NP 206-229) (0.2, 2 and 4 µg/ml). Eighteen hours before harvesting, 1 µCi of [^3^H] thymidine (Amersham International, Freiburg, Germany) was added to each well. Then, cells were harvested on paper filters (Filtermat A; Wallac, Freiburg, Germany) using a cell harvester (Inotech, Wohlen, Switzerland) and the incorporation of [^3^H] thymidine into the DNA of proliferating cells was determined using a scintillation counter (Wallac 1450, Micro-Trilux). The results are presented as stimulation index (SI). For the calculation of the SI the counts per minute (cpm) of antigen-stimulated samples (0.2, 2 and 4 µg/ml) were divided by cpm of unstimulated samples (0 µg/ml).

### Challenge

Groups (n = 6) of female BALB/c (H-2d) were immunized on day 0 and 21 with rNP (10 µg/dose) alone or co-administered with c-di-AMP (10 µg/dose) by intranasal route, whereas control animals received PBS. All groups were challenged on day 60 with a sub-lethal dose of the influenza strain A/Puerto Rico/8/34 (H1N1) (50 FFU/0.5 LD50). To this end, mice were anesthetized by intraperitoneal injection with Ketamine/xylazine combination with a dose adjusted to the individual body weight (200 µl/20 g). The virus was administered in a total volume of 20 µl, 10 µl per nostril. Following infection, mice were monitored daily for morbidity during two weeks.

### Statistical analysis

Statistical analysis was performed with GraphPad Prism (version 5.01), software. The data was presented as mean ±SEM. Difference between groups were analyzed by Student's t-test and one way ANOVA. Differences were considered significant at p<0.05.

## Results

### The immunization with rNP co-administered with c-di-AMP elicits strong antigen-specific humoral immune responses at systemic and mucosal levels

**Figure 1:**
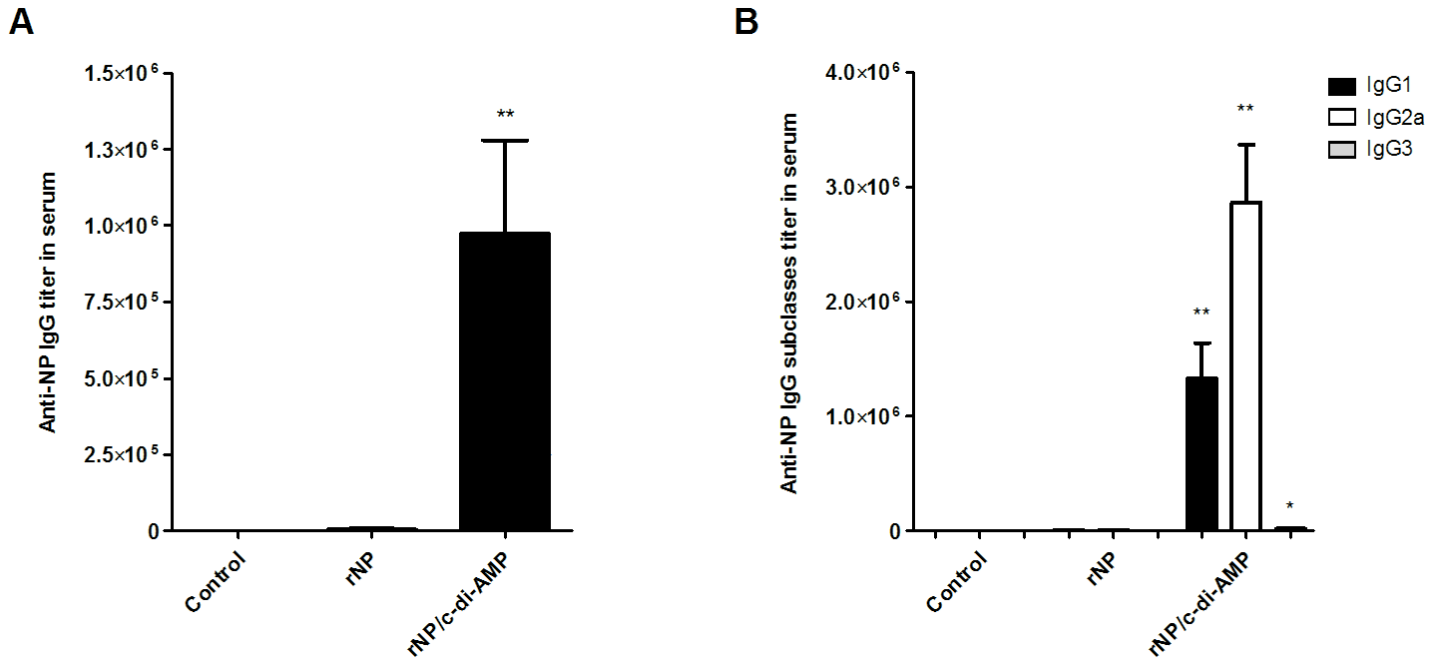
Evaluation of NP specific IgG and IgG subclasses in serum. Anti-NP IgG titers were determined by ELISA on day 60. BALB/c mice (n = 5) were vaccinated with two doses of rNP (10 µg/dose) alone or co-administered with c-di-AMP (10 µg/dose) by intranasal route, whereas control animals received PBS, at an interval of 3 weeks. (A) Anti-NP IgG titer. (B) Anti-NP IgG subclass titers. The results are expressed as mean end point titers. The S.E.M. is indicated by vertical lines. Differences were statistically significant at p<0.01 (**) or p<0.05 (*) with respect to values obtained in mice receiving the antigen alone.

**Figure 2:**
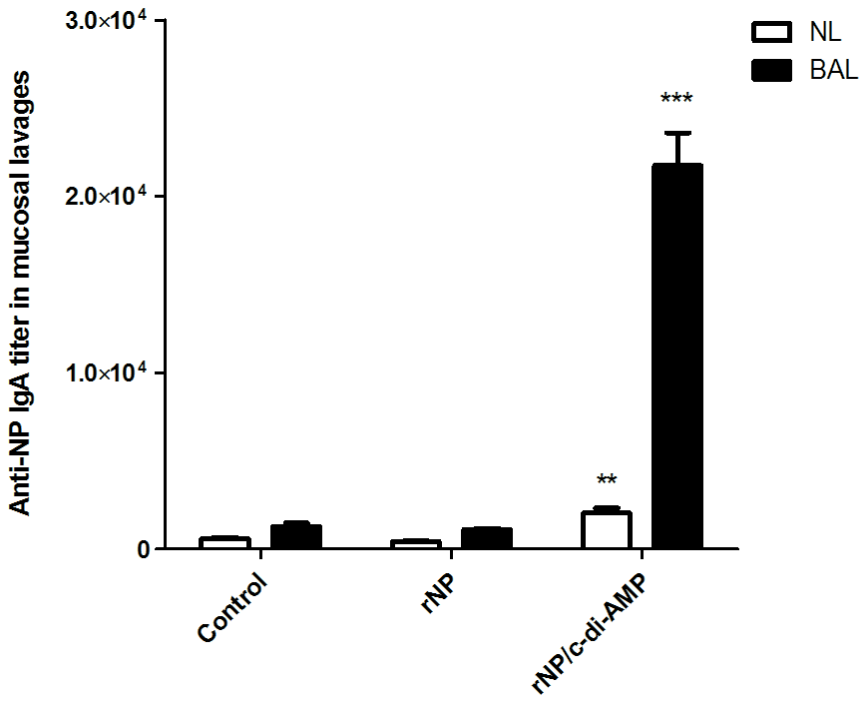
Evaluation of NP specific IgA in mucosal lavages. NP-specific IgA titers measured by ELISA in nasal (NL) and BAL lavages of BALB/c mice immunized with two doses of rNP (10 µg/dose) alone or co-administered with c-di-AMP (10 µg/dose) by intranasal route at an interval of 3 weeks. Control animals received PBS. Results are expressed as NP specific IgA titer. The S.E.M. is indicated by vertical lines. Differences were statistically significant at p<0.001 (***) and p<0.01 (**), compared with the titers in mice immunized with rNP alone.

Current influenza vaccines should be endowed with the capacity to induce an efficient humoral response, ideally, both at systemic and mucosal levels. We evaluated the capacity of rNP adjuvanted with c-di-AMP to induce humoral and cellular immune responses when given by intranasal route. NP was chosen as antigen due to the fact that this protein is one of the most promising candidates for the development of an influenza vaccine with broader cross-protective activity. In addition, rNP could be produced in *E. coli* at high yields and comparatively low costs [42]. To this end, groups of BALB/c mice were immunized by intranasal route with two doses of 10 µg of rNP alone, or co-administered with 10 µg of c-di-AMP, at an interval of three weeks. Immunized mice were bled six weeks after the second immunization and NP-specific total IgG and IgG subclass titers (e.g. IgG1, IgG2a and IgG3) were determined in serum by ELISA (Fig. 1). Sera of mice immunized with rNP co-administered with c-di-AMP showed high titers with an 239-fold increase of antigen-specific IgG, when compared with those from animals receiving rNP alone. The analysis of the IgG subclass titers showed that significantly enhanced NP specific IgG2a, IgG1, and IgG3 titers, with a preferential induction of the IgG2a subclass (IgG2a/IgG1 ratio 2.2) indicating a Th1 bias immune response. To evaluate the immune responses induced by the vaccine formulation at mucosal level, titers of NP-specific IgA in NL and BAL were determined by ELISA (Fig. 2). Similarly to what happened with the systemic response, strong responses were obtained by inclusion of c-di-AMP in the formulation. This is really important considering the fact that the primary replication site of influenza virus is the lung.

### Intranasal administration of rNP co-administered with c-di-AMP induces strong cellular immune responses

**Figure 3:**
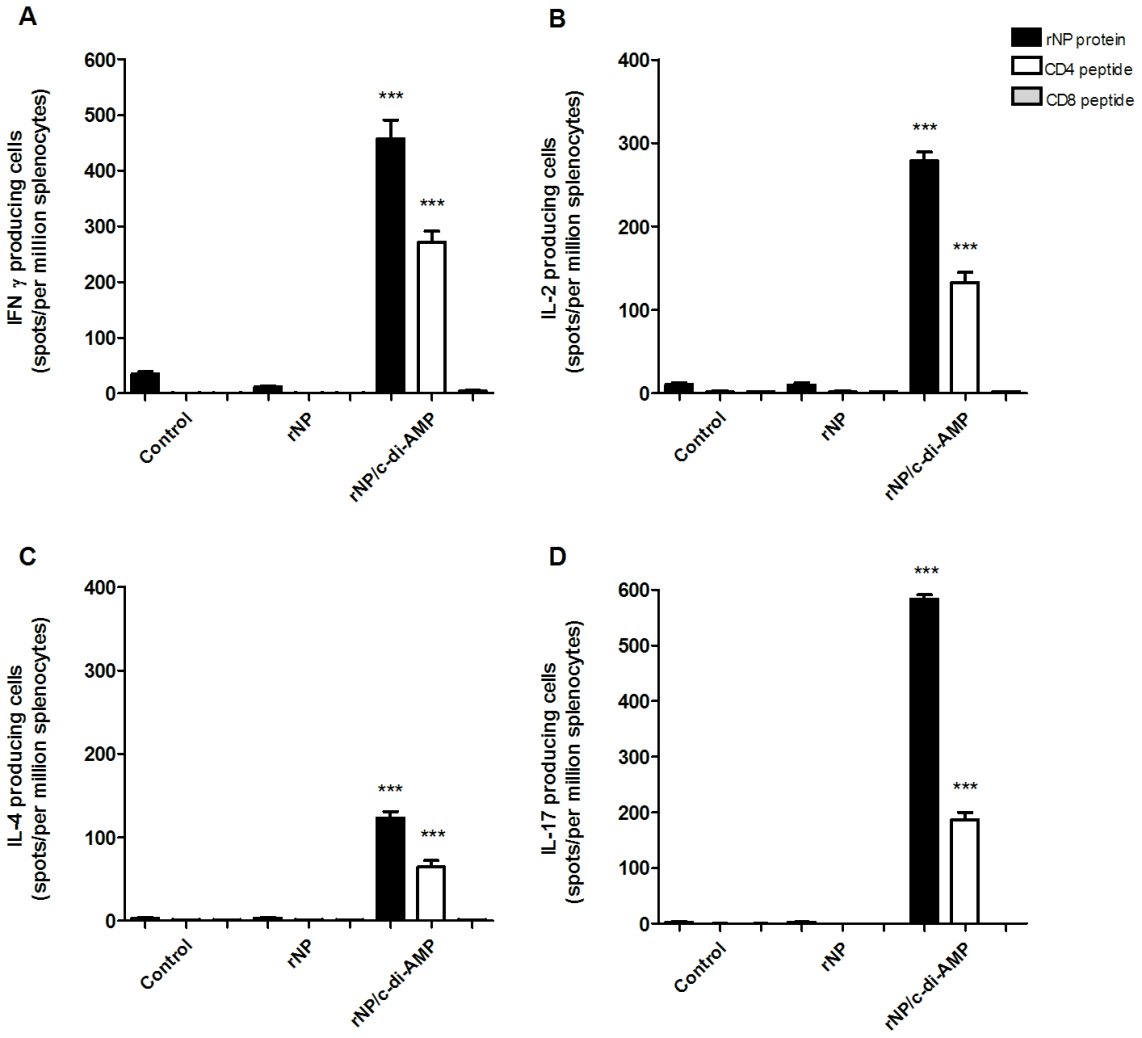
Analysis NP-specific cellular responses induced by vaccination. Splenocytes from immunized groups of mice were incubated for 24 or 48 h in the presence of rNP or peptides encompassing MHC class I and class II restricted epitopes of NP. The number of IFN-γ (A), IL-2 (B), IL-4 (C) and IL-17 (D) producing cells was then determined by ELISPOT. Results are expressed as number of spots of cells producing cytokines per 10^6^ spleen cells after subtraction of background values of unstimulated cells. The differences are statistically significant p<0.001 (***) compared to the results of cells obtained from mice vaccinated with rNP alone.

It has been previously reported that c-di-AMP is also capable of inducing a strong cellular response to co-administered antigens [41]. Thus, we evaluated if the novel NP vaccine formulation was also able to promote antigen-specific cellular responses. To this end, animals were sacrificed three weeks after the second immunization and the capacity of splenocytes to produce IL-2, INF-γ, IL-17 and IL-4 after re-stimulation with rNP or peptides corresponding to NP epitopes recognized in the context of MHC class I or class II molecules was determined by ELISPOT. In mice immunized with rNP co-administered with c-di-AMP strong cellular responses characterized by enhanced secretion of INF-γ, IL-2 and IL-17, whereas only a weak production of IL-4 was observed (Fig. 3), thereby suggesting the stimulation of a dominant Th1/Th17 response [41]. The analysis of T cell responses after re-stimulation with MHC class II or class I restricted peptides indicated that responses were mainly driven by CD4+ T cells.

**Figure 4:**
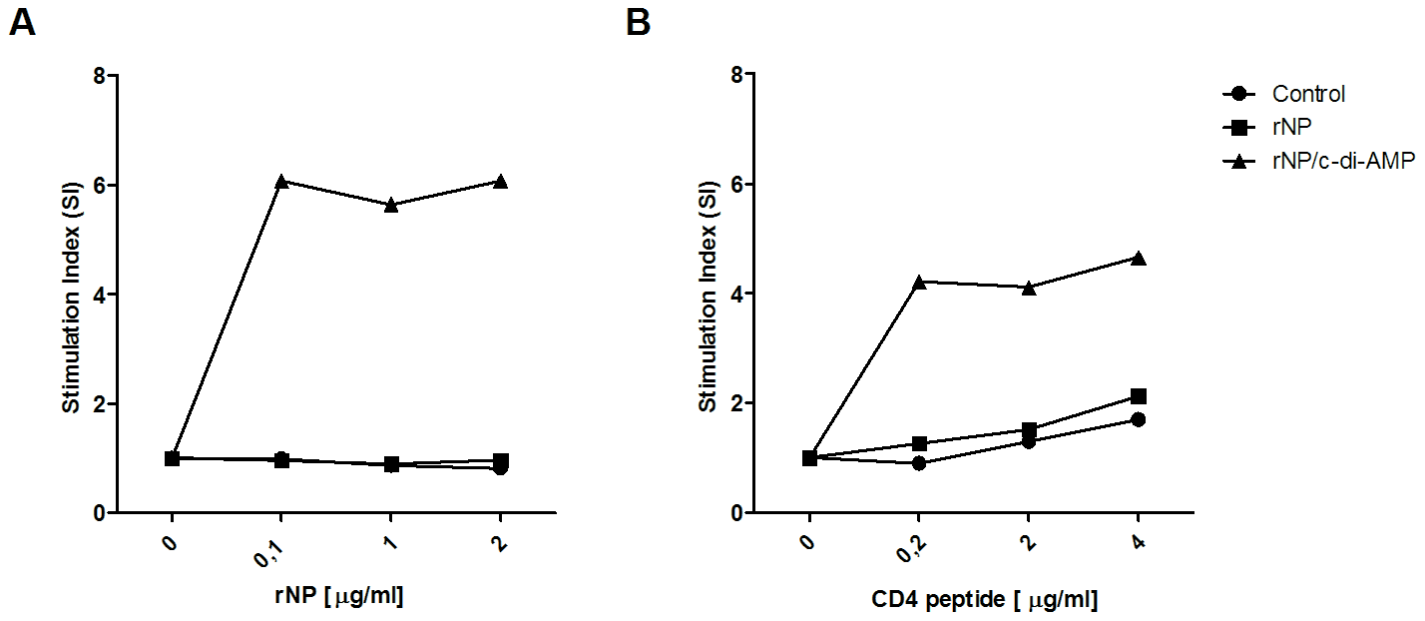
Analysis of proliferative responses in vaccinated mice. Splenocytes from immunized groups of mice were re-stimulated for 96 h with different concentrations of rNP (A) or a pool of peptides encompassing a MHC class II restricted epitopes of NP. Cellular proliferation was measured by incorporation of [^3^H] thymidine into the DNA. The results are represented as stimulation index (SI).

In addition, proliferative responses of splenocytes obtained from immunized mice after *in vitro* re-stimulation with rNP or peptides were evaluated (Fig. 4). Strong responses were observed in animals vaccinated with rNP co-administered with c-di-AMP. Moreover, strong proliferation was observed after stimulation with MHC class II peptides, thereby confirming that the cellular response is mainly driven by CD4+ T cells. In contrast, no or only weak stimulation was observed when spleen cells from mice vaccinated with rNP alone or PBS after were analyzed. This is in line with the ELISPOT results which also showed no or only weak stimulation when stimulated with a MHC class I specific peptide (data not shown).

### Mice vaccinated with rNP co-administered with c-di-AMP are protected against challenge with the H1N1 influenza virus

**Figure 5:**
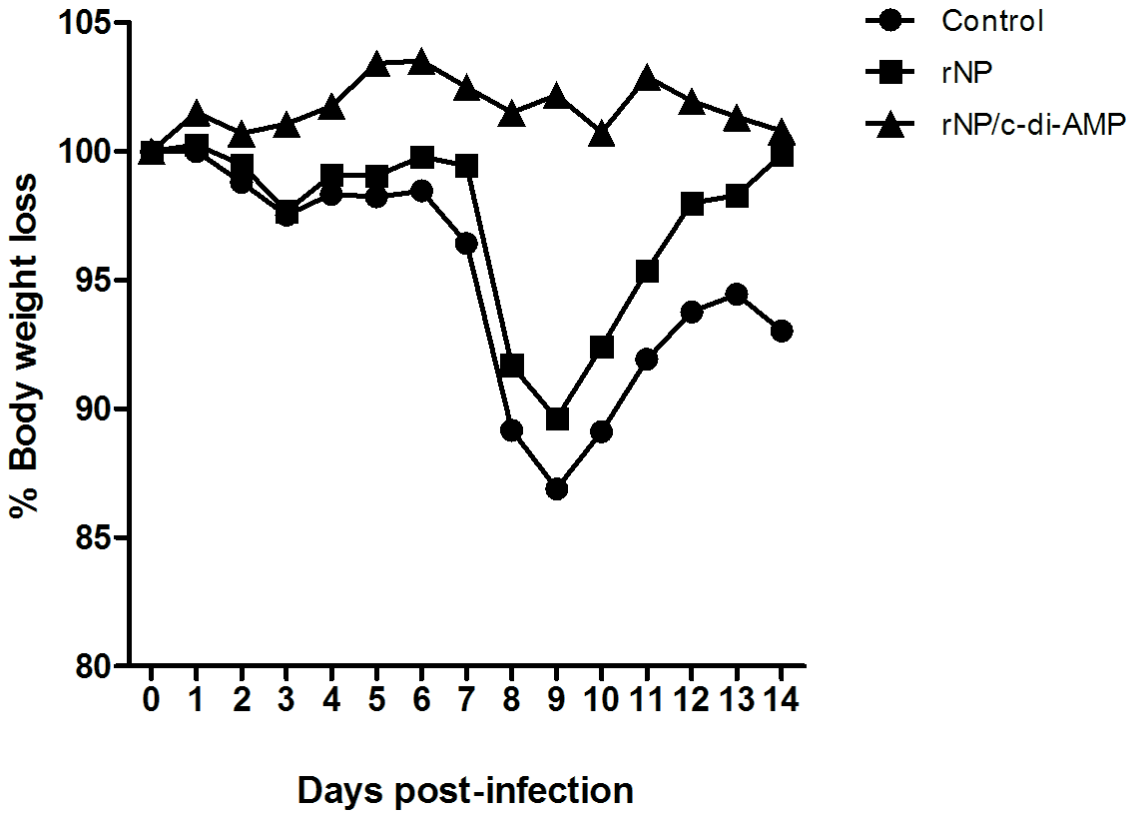
Protection of vaccinated mice against challenge with the influenza A virus. BALB/c mice (n = 6) were immunized on day 0 and 21 with rNP (10 µg/dose) alone or co-administered with c-di-AMP (10 µg/dose) by intranasal route, whereas control animals received PBS. All groups were challenged on day 60 with a non-lethal dose of the influenza strain A/Puerto Rico/8/34 (H1N1). Animal body weight was then monitored for 14 days. Results are expressed as average percentage of weight loss.

Mice vaccinated with rNP alone or co-administered with c-di-AMP were challenged at day 60 post priming with the influenza strain A/PR/8/34 (H1N1) and their body weight was recorded daily for at least two weeks as indicator for the ongoing infection disease (Fig. 5). From day 2 after challenge onwards, control mice and animals vaccinated with rNP alone started to develop signs of disease, such as loss of body weight, ruffled fur and lethargy. At days 8-9 post challenge, all mice of the control and rNP-alone groups showed signs of illness (e.g. ruffled fur and/or lethargy), indicated by a loss of 10 to 13% of their original body weight. In contrast, mice immunized with rNP co-administered with c-di-AMP showed no weight loss or side effects after challenge. Taken together, these results confirm that the immune responses induced by intranasal vaccination with rNP co-administered with c-di-AMP confer protection against influenza virus infection.

## Discussion

Current influenza vaccines have certain shortcomings, including their reduced capacity to induce cellular and mucosal immunity. Provision of such attributes would markedly improve the performance of existing vaccines. Therefore, different approaches have been explored to overcome these problems, such as the use of alternative routes of immunization. In this regard, mucosal administration represents an attractive approach to promote immune responses at the pathogen portal entry. However, purified antigens are *per se* poor immunogenic when administered by different mucosal routes (e.g. oral). This can be overcome by co-administration with appropriate mucosal adjuvants.

Previous studies from our group suggested that the cyclic di-nucleotides are potent mucosal adjuvants, which are also able to promote efficient and humoral and cellular immune responses when co-administered with the hemagglutinin of the influenza virus, including stimulation of multi-functional T cells [41], [43]–[48]. Thus, in the current study we assess the effectiveness of c-di-AMP adjuvant co-administered with a rNP-based subunit influenza vaccine. We demonstrated that intranasal administration of rNP/c-di-AMP vaccine results in enhanced humoral and cellular immune responses at both systemic and mucosal levels, which confer effective protection against influenza infection.

Increased serum levels of NP antigen-specific antibodies, were observed in mice vaccinated with rNP/c-di-AMP vaccine (Fig. 1). Although specific IgG-NP antibodies have no neutralizing activity, their importance should not be neglected. Several studies suggested that they could contribute significantly to protection against influenza infection by different antiviral mechanisms. These studies have shown that NP-containing immune-complexes released from infected cells could bind to Fc receptors on dendritic cells, thereby enhancing antigen presentation and subsequent viral clearance of infected cells by CD8+ T cells [49], [50]. Furthermore, it was demonstrated that vaccination strategies to boost existing anti-NP Abs levels, can contribute to long term hetero-subtype immunity [50]. Based on these attributes it was suggested that the capacity to stimulate anti-NP IgG may be a critical feature of a universal influenza vaccine [49].

Another prominent feature of the rNP/c-d-AMP vaccine was the induction of a vigorous NP specific IgA response in NL and BAL secretions. This is particularly important since the influenza virus is a respiratory pathogen, colonizing trachea, bronchi and pulmonary alveoli as sites of viral replication. It is tempting to speculate that this IgA might also contribute to cross-protection by a process known as intracellular neutralization. Intracellular neutralization of viruses is a process whereby IgA antibodies may neutralize viral replication by binding to newly synthesized viral proteins after being internalized in infected cells. It has been demonstrated that IgA can be internalized within epithelial cells by the polymeric immunoglobulin receptor (pIgR), and inside the cell it can be able to bind to newly synthesized viral proteins, preventing viral assembly and neutralizing viral infection. It was shown that this mechanism works with various viruses (e.g. influenza, sendai, measles, HIV, etc.) and it may be mediated by antibodies recognizing the envelope proteins as well as the internal proteins of the virus as is the case of influenza NP [51]–[56]. Another appealing attribute of the rNP/c-di-AMP vaccine is its extraordinary ability to induce a strong cellular response. Cytokine profiles of splenocytes from immunized mice showed a strong secretion of IL-2 and INF-γ compared to the values from mice receiving rNP alone (Fig. 3), thereby suggesting a Th1 dominating immune response.

In addition we showed, that splenocytes restimulated with NP are able to secrete remarkable IL-17 levels, which is consistent with previously reported results on the use of c-di-AMP as mucosal adjuvant [41]. The true role of IL-17 in influenza infection is still a matter of debate, with evidence supporting its contribution to both pathogenic or protective processes [57]–[61]. It has been indicated that IL-17 may have a beneficial role at the level of bronchial mucosa against respiratory pathogens through the induction of polymeric Ig receptor expression in the airway epithelium and by enhancing the secretory IgA levels [62]. It was also demonstrated that in IL-10-deficient mice there is an increased expression of several Th17 cytokines in the lungs and this correlates with a dramatically higher survival compared with wild-type mice when challenged with lethal doses of virus [58]. There is some evidence suggesting that the use of Th17 inducing adjuvants may result in increased morbidity and exacerbated lung injury upon subsequent influenza infection [63]. In our study no signs of exacerbate morbidity was observed in mice vaccinated with rNP/c-di-AMP post challenge. We showed that a balanced Th1/Th17 profile is important to influenza protection. Nevertheless, a better understanding of the role played by different subsets of IL-17 producing cells during influenza infection, as well as on the viral clearance or immune pathological mechanisms to which these cells contribute will be helpful to understand true impact of IL-17 regulation or dysregulation during infection and vaccination.

In addition, our results showed that the rNP/c-di-AMP vaccine induces potent CD4 response after stimulation with MHC class II NP restricted peptides. Recent reports have suggested the importance of memory CD4+ T cells in influenza infection, as a result of their synergistic helper functions with B or T cells, as well as their contribution to the elimination of escape viral mutants in absence of B cells or CD8+ T cells [31], [64], [65]. Thus, the development of vaccines able to trigger strong CD4+ responses could be central for the induction of memory responses capable of combating divergent influenza viruses through multiple pathways. In this context, there is experimental evidence on NP-based vaccines which promote CD4+ T cell responses contributing towards protective immunity [22], [66]. This phenomenon is not restricted only to influenza, since numerous infectious models have demonstrated the importance of CD4+ T cells in cellular mediated protection [67], [68].

Taken together, the quality of the immune response stimulated by intranasal administration with rNP/c-di-AMP vaccine was clearly demonstrated by the protection conferred by this formulation against a challenge with the influenza virus. This study clearly demonstrated that c-di-AMP is a promising mucosal adjuvant, which should be exploited to develop innovative vaccines against seasonal and pandemic influenza.

## References

[1] Belongia E, Kieke B, Coleman L, Donahue J, Irving S, et al (2008) Interim Within-Season Estimate of the Effectiveness of Trivalent Inactivated Influenza Vaccine — Marshfield, Wisconsin, 2007–08 Influenza Season. MMWR Morb Mortal Wkly Rep 18: 393–8. 18418344

[2] Osterholm MT, Kelley NS, Sommer A, Belongia EA (2012) Efficacy and effectiveness of influenza vaccines: a systematic review and meta-analysis. Lancet Infect Dis 12: 36–44. 22032844 10.1016/S1473-3099(11)70295-X

[3] Boyce TG, Hsu HH, Sannella EC, Coleman-Dockery SD, Baylis E, et al (2000) Safety and immunogenicity of adjuvanted and unadjuvanted subunit influenza vaccines administered intranasally to healthy adults. Vaccine 19: 217–226. 10930676 10.1016/s0264-410x(00)00171-7

[4] Brokstad KA, Eriksson J-C, Cox RJ, Tynning T, Olofsson J, et al (2002) Parenteral vaccination against influenza does not induce a local antigen-specific immune response in the nasal mucosa. J Infect Dis 185: 878–884. 11920311 10.1086/339710

[5] Ennis FA, Cruz J, Jameson J, Klein M, Burt D, et al (1999) Augmentation of human influenza A virus-specific cytotoxic T lymphocyte memory by influenza vaccine and adjuvanted carriers (ISCOMS). Virology 259: 256–261. 10388649 10.1006/viro.1999.9765

[6] Rimmelzwaan GF, Nieuwkoop N, Brandenburg A, Sutter G, Beyer WE, et al (2000) A randomized, double blind study in young healthy adults comparing cell mediated and humoral immune responses induced by influenza ISCOM vaccines and conventional vaccines. Vaccine 19: 1180–1187. 11137255 10.1016/s0264-410x(00)00310-8

[7] O'Donnell CD, Vogel L, Wright A, Das SR, Wrammert J, et al (2012) Antibody Pressure by a Human Monoclonal Antibody Targeting the 2009 Pandemic H1N1 Virus Hemagglutinin Drives the Emergence of a Virus with Increased Virulence in Mice. MBio 3: e00120–12–e00120–12 doi:10.1128/mBio.00120-12 22647789 10.1128/mBio.00120-12 PMC3372962

[8] Li CK, Rappuoli R, Xu X-N (2013) Correlates of protection against influenza infection in humans—on the path to a universal vaccine? Curr Opin Immunol 25: 470–476. 23948572 10.1016/j.coi.2013.07.005

[9] Fiers W, De Filette M, El Bakkouri K, Schepens B, Roose K, et al (2009) M2e-based universal influenza A vaccine. Vaccine 27: 6280–6283. 19840661 10.1016/j.vaccine.2009.07.007

[10] Margine I, Krammer F, Hai R, Heaton NS, Tan GS, et al (2013) Hemagglutinin stalk-based universal vaccine constructs protect against group 2 influenza A viruses. J Virol 87: 10435–10446. 23903831 10.1128/JVI.01715-13 PMC3807421

[11] Vitelli A, Quirion MR, Lo C-Y, Misplon JA, Grabowska AK, et al (2013) Vaccination to conserved influenza antigens in mice using a novel Simian adenovirus vector, PanAd3, derived from the bonobo Pan paniscus. PLoS One 8: e55435. 23536756 10.1371/journal.pone.0055435 PMC3594242

[12] Berthoud TK, Hamill M, Lillie PJ, Hwenda L, Collins KA, et al (2011) Potent CD8+ T-cell immunogenicity in humans of a novel heterosubtypic influenza A vaccine, MVA-NP+M1. Clin Infect Dis 52: 1–7. 21148512 10.1093/cid/ciq015 PMC3060888

[13] Lillie PJ, Berthoud TK, Powell TJ, Lambe T, Mullarkey C, et al (2012) Preliminary assessment of the efficacy of a T-cell-based influenza vaccine, MVA-NP+M1, in humans. Clin Infect Dis 55: 19–25. 22441650 10.1093/cid/cis327 PMC3369564

[14] Schotsaert M, Saelens X, Leroux-Roels G (2012) Influenza vaccines: T-cell responses deserve more attention. Expert Rev Vaccines 11: 949–962. 23002976 10.1586/erv.12.71

[15] Effros RB, Doherty PC, Gerhard W, Bennink J (1977) Generation of both cross-reactive and virus-specific T-cell populations after immunization with serologically distinct influenza A viruses. J Exp Med 145: 557–568. 233901 10.1084/jem.145.3.557 PMC2180700

[16] Yap KL, Ada GL, McKenzie IF (1978) Transfer of specific cytotoxic T lymphocytes protects mice inoculated with influenza virus. Nature 273: 238–239. 306072 10.1038/273238a0

[17] Yewdell JW, Bennink JR, Smith GL, Moss B (1985) Influenza A virus nucleoprotein is a major target antigen for cross-reactive anti-influenza A virus cytotoxic T lymphocytes. Proc Natl Acad Sci U S A 82: 1785–1789. 3872457 10.1073/pnas.82.6.1785 PMC397357

[18] Taylor PM, Askonas BA (1986) Influenza nucleoprotein-specific cytotoxic T-cell clones are protective in vivo. Immunology 58: 417–420. 2426185 PMC1453480

[19] McMichael AJ, Gotch FM, Noble GR, Beare PA (1983) Cytotoxic T-cell immunity to influenza. N Engl J Med 309: 13–17. 6602294 10.1056/NEJM198307073090103

[20] Kreijtz JHCM, de Mutsert G, van Baalen CA, Fouchier RAM, Osterhaus ADME, et al (2008) Cross-recognition of avian H5N1 influenza virus by human cytotoxic T-lymphocyte populations directed to human influenza A virus. J Virol 82: 5161–5166. 18353950 10.1128/JVI.02694-07 PMC2395172

[21] Grant E, Wu C, Chan K-F, Eckle S, Bharadwaj M, et al (2013) Nucleoprotein of influenza A virus is a major target of immunodominant CD8+ T-cell responses. Immunol Cell Biol 91: 184–194. 23399741 10.1038/icb.2012.78

[22] Tamura S, Miyata K, Matsuo K, Asanuma H, Takahashi H, et al (1996) Acceleration of influenza virus clearance by Th1 cells in the nasal site of mice immunized intranasally with adjuvant-combined recombinant nucleoprotein. J Immunol 156: 3892–3900. 8621928

[23] Guo L, Zheng M, Ding Y, Li D, Yang Z, et al (2010) Protection against multiple influenza A virus subtypes by intranasal administration of recombinant nucleoprotein. Arch Virol 155: 1765–1775. 20652335 10.1007/s00705-010-0756-3

[24] Rangel-Moreno J (2008) B cells promote resistance to heterosubtypic strains of influenza via multiple mechanisms. J Immunol 180: 454–463. 18097047 10.4049/jimmunol.180.1.454 PMC2712821

[25] Macleod MKL, David A, Jin N, Noges L, Wang J, et al (2013) Influenza nucleoprotein delivered with aluminium salts protects mice from an influenza A virus that expresses an altered nucleoprotein sequence. PLoS One 8: e61775. 23613928 10.1371/journal.pone.0061775 PMC3629017

[26] Huang B, Wang W, Li R, Wang X, Jiang T, et al (2012) Influenza A virus nucleoprotein derived from Escherichia coli or recombinant vaccinia (Tiantan) virus elicits robust cross-protection in mice. Virol J 9: 322. 23272943 10.1186/1743-422X-9-322 PMC3547759

[27] Savard C, Laliberté-Gagné MÈ, Babin C, Bolduc M, Guérin A, et al (2012) Improvement of the PapMV nanoparticle adjuvant property through an increased of its avidity for the antigen [influenza NP]. Vaccine 30: 2535–2542. 22326774 10.1016/j.vaccine.2012.01.085

[28] Patterson DP, Rynda-Apple A, Harmsen AL, Harmsen AG, Douglas T (2013) Biomimetic antigenic nanoparticles elicit controlled protective immune response to influenza. ACS Nano 7: 3036–3044. 23540530 10.1021/nn4006544 PMC3773536

[29] Zhong W, Liu F, Dong L, Lu X, Hancock K, et al (2010) Significant impact of sequence variations in the nucleoprotein on CD8 T cell-mediated cross-protection against influenza A virus infections. PLoS One 5: e10583. 20485501 10.1371/journal.pone.0010583 PMC2868023

[30] Hillaire MLB, Osterhaus ADME, Rimmelzwaan GF (2011) Induction of virus-specific cytotoxic T lymphocytes as a basis for the development of broadly protective influenza vaccines. J Biomed Biotechnol 2011: 939860. 22007149 10.1155/2011/939860 PMC3189652

[31] McKinstry KK, Strutt TM, Kuang Y, Brown DM, Sell S, et al (2012) Memory CD4+ T cells protect against influenza through multiple synergizing mechanisms. J Clin Invest 122: 2847–2856. 22820287 10.1172/JCI63689 PMC3408751

[32] Boonnak K, Subbarao K (2012) Memory CD4+ T cells: beyond “helper” functions. J Clin Invest 122: 2768–2770. 22820285 10.1172/JCI65208 PMC3408759

[33] Price GE, Soboleski MR, Lo CY, Misplon J, Quirion MR, et al (2010) Single-dose mucosal immunization with a candidate universal influenza vaccine provides rapid protection from virulent H5N1, H3N2 and H1N1 viruses. PLoS One 5: e13162. 20976273 10.1371/journal.pone.0013162 PMC2953831

[34] Sekaly RP (2008) The failed HIV Merck vaccine study: a step back or a launching point for future vaccine development? J Exp Med 205: 7–12. 18195078 10.1084/jem.20072681 PMC2234358

[35] Rose M, Zielen S, Baumann U (2012) Mucosal immunity and nasal influenza vaccination. Expert Rev Vaccines 11: 595–607. 22827245 10.1586/erv.12.31

[36] Lycke N (2012) Recent progress in mucosal vaccine development: potential and limitations. Nat Rev Immunol 12: 592–605. 22828912 10.1038/nri3251

[37] Holmgren J, Czerkinsky C (2005) Mucosal immunity and vaccines. Nat Med 11: S45–53. 15812489 10.1038/nm1213

[38] Falkeborn T, Bråve A, Larsson M, Akerlind B, Schröder U, et al (2013) Endocine, N3OA and N3OASq; three mucosal adjuvants that enhance the immune response to nasal influenza vaccination. PLoS One 8: e70527. 23950951 10.1371/journal.pone.0070527 PMC3738562

[39] Liu H, Patil HP, de Vries-Idema J, Wilschut J, Huckriede A (2013) Evaluation of mucosal and systemic immune responses elicited by GPI-0100- adjuvanted influenza vaccine delivered by different immunization strategies. PLoS One 8: e69649. 23936066 10.1371/journal.pone.0069649 PMC3729563

[40] Svindland SC, Pedersen GK, Pathirana RD, Bredholt G, Nøstbakken JK, et al (2013) A study of Chitosan and c-di-GMP as mucosal adjuvants for intranasal influenza H5N1 vaccine. Influenza Other Respi Viruses 7: 1181–1193. 10.1111/irv.12056 PMC4634239 23170900

[41] Ebensen T, Libanova R, Schulze K, Yevsa T, Morr M, et al (2011) Bis-(3′,5′)-cyclic dimeric adenosine monophosphate: strong Th1/Th2/Th17 promoting mucosal adjuvant. Vaccine 29: 5210–5220. 21619907 10.1016/j.vaccine.2011.05.026

[42] Cargnelutti DE, Sanchez M V, Alvarez P, Boado L, Glikmann G, et al (2012) Improved immune response to recombinant influenza nucleoprotein formulated with ISCOMATRIX. J Microbiol Biotechnol 22: 416–421. 22450799 10.4014/jmb.1106.06021

[43] Pedersen GK, Ebensen T, Gjeraker IH, Svindland S, Bredholt G, et al (2011) Evaluation of the sublingual route for administration of influenza H5N1 virosomes in combination with the bacterial second messenger c-di-GMP. PLoS One 6: e26973. 22069479 10.1371/journal.pone.0026973 PMC3206068

[44] Madhun AS, Haaheim LR, Nøstbakken JK, Ebensen T, Chichester J, et al (2011) Intranasal c-di-GMP-adjuvanted plant-derived H5 influenza vaccine induces multifunctional Th1 CD4+ cells and strong mucosal and systemic antibody responses in mice. Vaccine 29: 4973–4982. 21600260 10.1016/j.vaccine.2011.04.094

[45] Libanova R, Ebensen T, Schulze K, Bruhn D, Nörder M, et al (2010) The member of the cyclic di-nucleotide family bis-(3′, 5′)-cyclic dimeric inosine monophosphate exerts potent activity as mucosal adjuvant. Vaccine 28: 2249–2258. 20060510 10.1016/j.vaccine.2009.12.045

[46] Ebensen T, Guzmán CA (2009) Immune modulators with defined molecular targets: cornerstone to optimize rational vaccine design. Adv Exp Med Biol 655: 171–188. 20047042 10.1007/978-1-4419-1132-2_13

[47] Ebensen T, Schulze K, Riese P, Morr M, Guzmán C A (2007) The bacterial second messenger cdiGMP exhibits promising activity as a mucosal adjuvant. Clin Vaccine Immunol 14: 952–958. 17567766 10.1128/CVI.00119-07 PMC2044480

[48] Ebensen T, Schulze K, Riese P, Link C, Morr M, et al (2007) The bacterial second messenger cyclic diGMP exhibits potent adjuvant properties. Vaccine 25: 1464–1469. 17187906 10.1016/j.vaccine.2006.10.033

[49] LaMere MW, Lam H-T, Moquin A, Haynes L, Lund FE, et al (2011) Contributions of antinucleoprotein IgG to heterosubtypic immunity against influenza virus. J Immunol 186: 4331–4339. 21357542 10.4049/jimmunol.1003057 PMC3159153

[50] Lamere MW, Moquin A, Lee FE-H, Misra RS, Blair PJ, et al (2011) Regulation of antinucleoprotein IgG by systemic vaccination and its effect on influenza virus clearance. J Virol 85: 5027–5035. 21367900 10.1128/JVI.00150-11 PMC3126167

[51] Mazanec MB, Kaetzel CS, Lamm ME, Fletcher D, Nedrud JG (1992) Intracellular neutralization of virus by immunoglobulin A antibodies. Proc Natl Acad Sci U S A 89: 6901–6905. 1323121 10.1073/pnas.89.15.6901 PMC49612

[52] Mazanec MB, Coudret CL, Fletcher DR (1995) Intracellular neutralization of influenza virus by immunoglobulin A anti-hemagglutinin monoclonal antibodies. J Virol 69: 1339–1343. 7815518 10.1128/jvi.69.2.1339-1343.1995 PMC188717

[53] Aiyegbo MS, Sapparapu G, Spiller BW, Eli IM, Williams DR, et al (2013) Human rotavirus VP6-specific antibodies mediate intracellular neutralization by binding to a quaternary structure in the transcriptional pore. PLoS One 8: e61101. 23671563 10.1371/journal.pone.0061101 PMC3650007

[54] Zhou D, Zhang Y, Li Q, Chen Y, He B, et al (2011) Matrix protein-specific IgA antibody inhibits measles virus replication by intracellular neutralization. J Virol 85: 11090–11097. 21865386 10.1128/JVI.00768-11 PMC3194966

[55] Corthésy B, Benureau Y, Perrier C, Fourgeux C, Parez N, et al (2006) Rotavirus anti-VP6 secretory immunoglobulin A contributes to protection via intracellular neutralization but not via immune exclusion. J Virol 80: 10692–10699. 16956954 10.1128/JVI.00927-06 PMC1641769

[56] Bomsel M, Heyman M, Hocini H, Lagaye S, Belec L, et al (1998) Intracellular neutralization of HIV transcytosis across tight epithelial barriers by anti-HIV envelope protein dIgA or IgM. Immunity 9: 277–287. 9729048 10.1016/s1074-7613(00)80610-x

[57] Li C, Yang P, Sun Y, Li T, Wang C, et al (2012) IL-17 response mediates acute lung injury induced by the 2009 pandemic influenza A (H1N1) virus. Cell Res 22: 528–538. 22025253 10.1038/cr.2011.165 PMC3292301

[58] McKinstry KK, Strutt TM, Buck A, Curtis JD, Dibble JP, et al (2009) IL-10 deficiency unleashes an influenza-specific Th17 response and enhances survival against high-dose challenge. J Immunol 182: 7353–7363. 19494257 10.4049/jimmunol.0900657 PMC2724021

[59] Hamada H, Garcia-Hernandez M de la L, Reome JB, Misra SK, Strutt TM, et al (2009) Tc17, a unique subset of CD8 T cells that can protect against lethal influenza challenge. J Immunol 182: 3469–3481. 19265125 10.4049/jimmunol.0801814 PMC2667713

[60] Wang X, Chan CCS, Yang M, Deng J, Poon VKM, et al (2011) A critical role of IL-17 in modulating the B-cell response during H5N1 influenza virus infection. Cell Mol Immunol 8: 462–468. 21946434 10.1038/cmi.2011.38 PMC4012931

[61] Kudva A, Scheller E V, Robinson KM, Crowe CR, Choi SM, et al (2011) Influenza A inhibits Th17-mediated host defense against bacterial pneumonia in mice. J Immunol 186: 1666–1674. 21178015 10.4049/jimmunol.1002194 PMC4275066

[62] Jaffar Z, Ferrini ME, Herritt LA, Roberts K (2009) Cutting edge: lung mucosal Th17-mediated responses induce polymeric Ig receptor expression by the airway epithelium and elevate secretory IgA levels. J Immunol 182: 4507–4511. 19342622 10.4049/jimmunol.0900237 PMC2740792

[63] Gopal R, Rangel-Moreno J, Fallert Junecko BA, Mallon DJ, Chen K, et al (2014) Mucosal pre-exposure to Th17-inducing adjuvants exacerbates pathology after influenza infection. Am J Pathol 184: 55–63. 24183780 10.1016/j.ajpath.2013.09.012 PMC3873493

[64] Kai McKinstry K, Dutton RW, Swain SL, Strutt TM (2013) Memory CD4 T Cell-Mediated Immunity against Influenza A Virus: More than a Little Helpful. Arch Immunol Ther Exp (Warsz) 61(5): 341–53. 23708562 10.1007/s00005-013-0236-z PMC3874125

[65] Wilkinson TM, Li CKF, Chui CSC, Huang AKY, Perkins M, et al (2012) Preexisting influenza-specific CD4+ T cells correlate with disease protection against influenza challenge in humans. Nat Med 18: 274–280. 22286307 10.1038/nm.2612

[66] Wang W, Huang B, Jiang T, Wang X, Qi X, et al (2012) Robust immunity and heterologous protection against influenza in mice elicited by a novel recombinant NP-M2e fusion protein expressed in E. coli. PLoS One 7: e52488. 23285063 10.1371/journal.pone.0052488 PMC3528677

[67] Hogan RJ, Zhong W, Usherwood EJ, Cookenham T, Roberts AD, et al (2001) Protection from respiratory virus infections can be mediated by antigen-specific CD4(+) T cells that persist in the lungs. J Exp Med 193: 981–986. 11304559 10.1084/jem.193.8.981 PMC2193400

[68] Sun K, Ye J, Perez DR, Metzger DW (2011) Seasonal FluMist vaccination induces cross-reactive T cell immunity against H1N1 (2009) influenza and secondary bacterial infections. J Immunol 186: 987–993. 21160043 10.4049/jimmunol.1002664

